# CML Developing Three Years after Therapy Completion in a Child with Ph(−) Pre-B-ALL

**DOI:** 10.1155/2011/670603

**Published:** 2011-09-21

**Authors:** S. Hosseini, G. H. Bahoush, P. Vousogh, Sh. Ansari, Kh. Arjmandi, M. Sabzechian

**Affiliations:** ^1^Hematology Laboratory, Hematology Oncology Ward, Ali Asghar Children Hospital, Tehran, Iran; ^2^Tehran University of Medical Science, Tehran, Iran

## Abstract

We report a girl with a history of Ph(−) pre-B-ALL and three years of disease-free survival admitting to our hospital for regular end of treatment checkup with an increased white blood cell count which in follow-up studies and molecular detection of BCR-ABL (p210) fusions gene had been diagnosed as a Ph(+) typical CML. The upcoming question in this case scenario is whether developed CML is a secondary leukemia due to previous ALL chemotherapy or just a relapse case of primary leukemia.

## 1. Introduction

Chronic myeloid leukemia (CML) is a Clonal Myeloproliferative expansion of transformed, primitive hematopoietic progenitor cells [1].

The Philadelphia (Ph) chromosome is the hallmark of CML and the most frequent cytogenetic abnormality known in human leukemia's and can be detected in more than 95% of patients with chronic myeloid leukemia (CML) [2], in a range of 20% to 40% of adults with acute lymphoblastic leukemia (ALL), 2% to 5% of children with ALL, and in rare cases of acute myelogenous leukemia [3–5]. 

The Ph chromosome is a shortened chromosome 22 resulting from a reciprocal translocation, t (9; 22)-(q34; q11), between the long arms of chromosomes 9 and 22 [6]. 

The cytogenetic lesion in Ph(+) CML and Ph(+) ALL has been shown to differ at the molecular level. In CML, the translocation is almost always associated with a breakpoint on Chromosome 22 that generally is known as the major breakpoint cluster region (M-BCR). This gives rise to a (P210) fusion protein in contrast to most cases of ALL which the break point is in the minor cluster region (m-BCR) and gives rise to (P190) fusion protein [7].

 In pediatric Ph(+) ALL, nearly 90% of cases have the (p190) variant,but in adult ALL approximately 25% to 50% of cases have (p210) variant [8]. 

This specific chromosome translocation t (9; 22) (q34; q11), molecularly, generates the *BCR-ABL* fusion gene encoding a BCR-ABL protein with constitutively enhanced tyrosine kinase activity. The activated BCR-ABL tyrosine kinase has been shown to be a key factor in leukemic transformation in patients as in animal model or *in vitro* reviewed in Faderl et al. [9]. 

## 2. Case Report

 A 12-year-old girl with a history of fever, fatigue, and pancytopenia had been referred to Ali Asghar's Children Hospital from a clinic in Varamin (Tehran's suburb) on September 2003.

She had been admitted in infectious disease ward for workup of fever, pancytopenia, hepatosplenomegaly, and inguinal and cervical lymphadenopathy. The results of first CBC taken in Ali Asghar's laboratory were as follows. 

WBC: 1∗10^3^/*μ*L, RBC: 3.33 mil/uL, Hemoglobin: 8.7 g/dL, Platelet count: 54∗10^3^/*μ*L, Hematocrit: 20.1%, M.C.V: 78.9 fL, M.C.H: 26.3 pg, M.C.H.C: 33.6 g/dL.

She had been taking antibiotic, and the bacterial culture results were all negative at the time of admission. Upon consultation with hematology oncology ward, a Bone Marrow aspiration (BM) had been scheduled, and on microscopic evaluation a hypercellular BM with complete replacement by immature lymphocytes resembling L1-FAB subtype with negative myeloperoxidase cytochemistry were observed.

Bone Marrow samples had been sent to cytogenetic laboratory for karyotyping and molecular diagnosis of BCR-ABL fusion gene, and the results had been normal karyotype and negative for BCR-ABL (p210) and (p190) fusion gene.

A sample had also been sent to Iranian blood transfusion laboratory for Immunophenotyping and the results were as follows:

CD10^+^, CD19^+^, CD20^+^, HLA DR^+^ CD13^−^, and CD33^−^ and the interpretation were consistent with a pre-B-ALL. After diagnosis on April 2003, the patient had been treated according to the children ALL-BFM conventional protocol (without high dose methotrexate) with prophylactic cranial irradiation (1800 Rad). She had a good response to prednisolone and on day 28 BM aspiration had been consistent with hematological remission (<5% blasts), and the results of immunophenotyping had been normal. She had no serious complication during and in between chemotherapy cycles except for few leukopenic episodes that were corrected by G-CSF therapy. 

She had completed the whole protocol on January 2007 with BM at complete remission, normal CSF, normal Immunophenotyping, and normal uterus and ovaries sonographies. CBC results at completion of treatment were as follows.

WBC: 5.7∗10^3^/*μ*L, RBC: 3.47 mil/uL, Hemoglobin: 11.6 g/dL, Platelet count: 4∗10^3^/*μ*L, Neutrophile: 67%, lymphocyte: 30%, Monocyte: 3%. 

She stayed 3 years out of treatment and at complete remission and had regular visits, and on August 2008 she came for regular checkups and the results of her CBC were as follows. 

WBC: 69.9∗10^3^/*μ*L, 3.79 mil/uL, Hemoglobin: 11.3 g/dL, Hematocrit: 33.6%, M.C.V: 88.7 fL, M.C.H: 29.8 Pg, M.C.H.C: 33.6 g/dL, Platelet count: 136∗10^3^/*μ*L, Neutrophile: 60%, Eosinophile: 3%, promyelocyte: 2%, Band: 20%. 

 On physical examination a mild to moderate splenomegaly had been noticed, and she had been put on antibiotic in case of underlying infection, and bone marrow samples had been taken for cytogenetic, molecular and immunophenotyping evaluations and another CBC sample had been also taken on October 2008 and the results were as follows.

WBC: 82.6∗10^3^/*μ*L, RBC: 366 mil/uL, Hemoglobin: 10.9 g/dL, Hematocrit: 32.4%, M.C.V: 88.5 fL, M.C.H: 29.8 Pg, M.C.H.C: 33.6 g/dL, Platelet count: 152∗10^3^/*μ*L, Neutrophile: 51%, Eosinophile: 2%, Lymphocyte: 16%, Band: 27%, Promyelocyte: 1%, Myelocyte: 2%.

The results of molecular evaluation of BM aspirate had been reported negative for t (4, 11), t (1, 19), and t (12, 21) and negative for BCR-ABL (P190) fusion gene and positive for BCR-ABL (P210) fusion gene (TaqMan Technology), and cytogenetic results were positive for Ph-chromosome or t (9; 22).

She had been put on imatinib mesylate (Gleevec formerly ST-1571), and there had been a good hematologic and molecular response, and white blood cell count decreased from 76700/uL on April 2008 to 2400/uL on October 2008, and the BCR-ABL fusion gene decreased from 11700 cpn on May 2008 to undetectable on July 2010. On her last visit on April 2011, she had been under good control no organomegaly, stable condition and still receiving Gleevec, and her CBC results were as follows. 

WBC: 4.9∗10^3^/*μ*L, RBC: 3.38 mil/uL, Hemoglobin: 10.4 g/dL, Hematocrit: 31.7%, M.C.V: 93.8 fL, M.C.H.C: 32.8 g/dL, Platelet count: 172∗10^3^/*μ*L, Neutrophile: 63%, Eosinophile 1%, lymphocyte: 33.2, Monocyte: 3%.

According to the CBC results, the patient had been in complete hematologic remission and on molecular evaluation of BCR-ABL fusion gene; the patient had also been in complete molecular response (CMR) status (Table 1).

## 3. Discussion

 We describe here a girl with negative Ph(−) pre-B-ALL diagnosed with Ph(+) CML 3 years after completion of therapy and disease-free survival.

 The main question in this case is whether her leukemia had been secondary to the treatment received or had been actually a relapse of primary leukemia, but since the primary pre-B-ALL in this patient had been Ph(−) the developing CML could be a secondary leukemia most probably a consequence of prior treatment or other environmental exposures that eventually evolved to Ph(+) CML.

Second primary cancers represent an important complication of modern chemotherapy and radiotherapy. Therapy-related (tr) leukemias are among the most common second malignancies in both pediatric and adult populations. Whereas a reasonable amount of data is available regarding the epidemiology, molecular pathogenesis, clinical behavior and response to therapy of second primary acute leukemia, very little is known about therapy-related chronic myeloid leukemia (tr-CML).

 A better characterization of this entity could increase our understanding about the mechanisms of carcinogenesis, specially the induction of specific genetic abnormalities, for example, BCR-ABL fusion, following chemotherapy and/or radiotherapy exposure, could facilitate the investigation of the kinetics of the development of CML and also provide a model to study molecular events that might precede its development. 

The cancer-predisposing syndromes, the detection of BCR-ABL transcripts in healthy individuals, and the *in vitro* induction of BCR-ABL fusions by ionizing radiation are all discussed in the context of tr-CML. Finally, the need for a large epidemiological study to specifically assess the risk of developing second primary CML after chemotherapy and/or radiotherapy is stressed.

Another question that might still be in our mind is that, whether the results of Philadelphia chromosome cytogenetic analysis and molecular diagnosis of BCR-ABL had been underestimated due to technical defect at diagnosis and patient was actually Ph(+) ALL and had been misdiagnosed at first, but from the fact that Ph(+) ALL patients have a poor prognosis and the 5-year event-free survival (EFS) ranges from 10% to 20%, in Ph(+), versus 76% in the Ph(−) population and recurrent disease in these patients is mainly due to failure of treatment, and the poor prognosis persists even after stratification in a high-risk treatment protocol [7]. In our case due to the good response to prednisolone and being on conventional BFM protocol and completing the protocol without any complications and 3 years of disease free survival actually confirms Ph(−) ALL with a favorable prognosis. 

## 4. Conclusion 

This case may be the first case ever reported with such a unique presentation, which indicates strongly the need for long-term followup of patients, and, due to a history of Ph(−) pre-B-ALL and by the fact that CML is a molecular disease, to wait and watch, whether the natural history of CML molecular behavior is changed by the time and whether in this case whose patient had been taking imatinib (BCR-ABL tyrosine kinase inhibitor) since CML diagnosis, the appearance and persistence of cytogenetically abnormal Ph(−) clone arising in the context of imatinib-induced major molecular response is sustained or there might be some clonal progression and imatinib resistance in the future.

Another important point in this case is to follow up patient in the future and to see whether depletion of Ph(+) stem cells make hematopoesis dependent on Ph(−) stem cells, in theory nonmalignant, and eventually clonal changes of the MDS type or of acute leukemia may appear which may be representative of a preleukemic stage of Ph(−) CML with some level of genetic instability.

## Figures and Tables

**Table 1:** Presence of BCR-ABL transcript in sequential Bone Marrow samples.

Date	Status of the disease	BCR-ABL copy number
November 2008	CP	11700
August 2009	CHR	30
February 2010	MMR	3
July 2010	CMR	UN detectable

CP: chronicphase, (CHR): complete hematology remission, MMR: major molecular response, CMR: complete molecular response.
